# All Hazards Great and Small: Applying Disaster Risk Reduction to Environmental Justice Communities in South Carolina

**DOI:** 10.1029/2025GH001370

**Published:** 2026-03-17

**Authors:** Daniel J. Kilpatrick, Keisha D. Long, Omar Muhammad, Dwayne E. Porter, Paul A. Sandifer, Karen Sprayberry, Katya Altman, Sean Briggs, Brooke Brittain, Nancy Button, John A. Cooper, Jeremy Cothran, Beata Dewitt, Tatiana DiSalvo, Herbert Fraser‐Rahim, Chaquetta Greene, Gary Harris, Kimberly Washok‐Jones, Sheila Kimble, Merrie Koester, Nikhil Kulkarni, Herbert Maybank, Michael McGirr, Jude Owoh, Robert Reese, Michele Schaafsma, Judith Day (Taylor), Heath Kelsey, Namita Koppa, Daphne Wilson

**Affiliations:** ^1^ Arnold School of Public Health University of South Carolina Columbia SC USA; ^2^ South Carolina Department of Environmental Services Columbia SC USA; ^3^ Lowcountry Alliance for Model Communities North Charleston SC USA; ^4^ Center for Coastal Environmental and Human Health College of Charleston Charleston SC USA; ^5^ College of Behavioral Social and Health Sciences Clemson University Clemson SC USA; ^6^ Rosemont Community Charleston SC USA; ^7^ Center for Sustainable Communities Atlanta GA USA; ^8^ Lower Richland Community Richland County SC USA; ^9^ University of South Carolina Center for Science Education Charleston SC USA; ^10^ VI Forward Bristol VA USA; ^11^ Welltivity Franklin TN USA; ^12^ University of Maryland Center for Environmental Science Annapolis MD USA; ^13^ College of Arts and Sciences University of South Carolina Columbia SC USA; ^14^ U.S. Environmental Protection Agency Atlanta GA USA

**Keywords:** disaster, risk, reduction, environmental, justice, resilience

## Abstract

Community‐managed disaster risk reduction (CMDRR) puts communities at the center of disaster readiness by assessing hazards, vulnerabilities and capacities, conducting risk analyses, and implementing comprehensive disaster risk reduction (DRR) plans. The EJ Strong pilot program was established to increase the resilience of participating environmental justice (EJ) communities in South Carolina (SC). This was accomplished through a CMDRR training program focused on EJ communities in SC. The curriculum was based on training manuals developed for use in low‐ and middle‐income countries. We modified the curriculum to make it culturally relevant, cope with the ongoing COVID‐19 pandemic, and emphasize the application of training during field exercises within communities. In total, ∼110 community participants representing EJ communities across SC attended one or more of the workshops, virtual mini‐workshops, and/or field practicums. Invited speakers and program team members raised the overall total to ∼150, and 46 received certificates in CMDRR training after the final workshop. To the best of our knowledge, this is the first application of CMDRR training in the U.S. as well as the first focused on EJ communities. Other outcomes from EJ Strong included a state‐wide emergency food access map, a mobile‐enabled disaster risk assessment app, an internet‐based course that includes the CMDRR curriculum, incorporation of DRR materials for use in public school classrooms, air quality and flood monitoring systems for communities, and acquisition of follow‐on funding for communities and the program. Participant evaluations revealed high levels of satisfaction with and appreciation of the program's content.

## Plain Language Summary

The EJ Strong pilot program was developed to address disaster resilience capacity gaps in EJ communities across SC, especially those related to the ongoing burden of the COVID‐19 pandemic. We adapted a training in CMDRR developed for international settings to be culturally relevant and applicable to EJ communities in SC. Overall, more than 110 community participants representing EJ communities attended one or more of the training sessions. Program team members and invited speakers raised the overall total to ∼150. 46 received certificates in CMDRR training. To the best of our knowledge, this is the first application of CMDRR training in the U.S. and the first focused on EJ communities. Outcomes from the pilot include a mobile‐enabled disaster assessment app, an emergency food access map, follow‐on funding for resilience projects in EJ communities, among others. Participant evaluations revealed high levels of satisfaction with and appreciation of the program's content.

## Key Points

- A Disaster Risk Reduction pilot program was tested in U.S. Environmental Justice communities to address acute and chronic hazard risks
- We contextualized and applied an international curriculum in Community Managed Disaster Risk Reduction to U.S. EJ settings
- The result was a product that was holistic, geographically transferrable, and provided a solution to address disaster risks in the U.S

## 1 Introduction

Globally, over the last 100+ years, numbers and impacts of disasters increased markedly (Number of recorded natural disaster events, 2024). Undoubtedly, some of the increase is due to better reporting, but certainly not all. When the top 50 mega‐disasters, such as early 20th century famines, each of which resulted in deaths of >1 million people are excluded, overall mortalities appear to have risen with the growing numbers of non‐mega disasters (CRED Publication—2023 Disasters in Numbers—A Significant Year of Disaster Impact | UN‐SPIDER Knowledge Portal, 2023). These data do not include the recent COVID‐19 mega‐disaster, which accounted for some 7 million deaths worldwide (COVID‐19 Deaths | WHO COVID‐19 Dashboard, 2024).

In the U.S., the National Oceanic and Atmospheric Administration (NOAA) has reported the annual number of weather or climate‐related disasters that caused Consumer Price Index (CPI)‐adjusted economic losses of >US $1 billion since 1980 (Smith, 2025). The highest number of billion‐dollar disasters previously recorded, 22, occurred in 2020, with 20 in 2021, 18 in 2022, 28 (a significant new high) in 2023 and 24 as of 1 November 2024 (Smith, 2025). The annual average for the 1980–2023 period is 8.5 such events, while that for the most recent 5 years (2019–2023) is 20.4 (Smith, 2025).

The UN's Hyogo Framework for Action (2005) and Sendai Framework for Disaster Risk Reduction (2015) signaled a paradigm shift in the way governments deal with disasters, moving from managing disaster impacts after the fact to proactively minimizing disaster risks beforehand using an “all of society approach” (UNDRR, 2023). Considering the escalating impacts of disasters, such an approach is appropriate and necessary.

Disaster risk reduction (DRR) is “the concept and practice of reducing disaster risks through systematic efforts to analyze and manage disaster‐causing factors including reduced exposure to hazard threats, reduction of vulnerability of populations and property, wise management of land and environment, and increased preparedness for adverse events” (Tahir et al., 2022). Disasters are hazard events that exceed the capacity of affected communities to cope with them, resulting in disruptions to life and livelihoods, loss of life, property, and critical infrastructure, and physical and psychological injuries.

Disaster risk can be lessened by reducing hazards and vulnerabilities and increasing coping capacity in communities (Imperiale & Vanclay, 2024). This can be accomplished through Community Managed Disaster Risk Reduction (CMDRR) efforts where communities direct, manage, and control DRR activities so that they have the most beneficial effects at the community level. In contrast to top‐down approaches, CMDRR and the similarly entitled Community Based Disaster Risk Management (CBDRM) methods, ensure that communities not only participate in DRR practices but own the results (McLennan, 2020). CMDRR enables communities to control how they will reduce their exposure to hazards, decrease vulnerability of members and assets, and increase disaster coping capacity.

Increasing probabilities of accelerated sea level rise, extreme weather events including heatwaves and storms, catastrophic flooding, pandemics, war and other traumas (Becker et al., 2023; Dangendorf et al., 2023; Sandifer & Scott, 2021) highlight the need for CMDRR. Also, disasters tend to impact poor people and disadvantaged communities most heavily (Abramson et al., 2010; Arias & Xu, 2022; Sandifer, 2022) and growing threats from infectious microbes, pollution, and harmful algal blooms further complicate the hazard landscape for many communities (Berdalet et al., 2023; Chen et al., 2023; Gin et al., 2023; Landrigan et al., 2020; Scott et al., 2019).

Olson et al. (2020) eloquently stated the imperative as follows: “As hazard event losses mount around the world, sometimes horrifically, the need to bring events down from catastrophes and disasters to less damaging and more manageable emergencies is literally a life and death issue, and the key to that de‐escalation of event impacts is more effective DRR.” While significant progress has been made in numerous areas of the world since the Sendai Declaration (UNDRR, 2023), relatively little attention has been paid to community DRR in the U.S. Here we describe our experiences with CMDRR focused on environmental justice (EJ) communities in the U.S. and the potential for broader application of the approach across the U.S. and elsewhere.

Environmental Justice means “the fair treatment and meaningful involvement of all people regardless of race, color, national origin, or income, with respect to the development, implementation, and enforcement of environmental laws, regulations, and policies” (US EPA, 2024). Communities experiencing environmental injustices, especially those whose exposure to pollution, toxins, and other hazards is disproportionately high, are self‐identified and traditionally term themselves as EJ communities, fenceline communities (because they share boundary lines with industry or transportation routes), among other terms (Gochfeld & Burger, 2011; Johnston & Cushing, 2020). Often combined with their higher levels of exposure is the lack of meaningful involvement of residents in these communities in the decision‐making process of land use practices, zoning, etc. (Konisky, 2016). The work summarized herein is a combination of EJ and CMDRR efforts and how they overlap to address the risks of natural hazards and inherently includes technological hazards and climate change‐induced events. As training co‐developers, we attempted to be “all‐hazards” aware, while engaging communities to prioritize hazards according to their risks. The context also encompasses how risks from various hazard types can lead to disaster, especially in communities that are most proximate to such events, and which have a lesser capacity to mitigate, prepare for, respond to, and recover from them.

The EJ Strong program, formally entitled “EJ Strong: Strengthening Communities for Disaster Risk Reduction, Response & Recovery in South Carolina,” was established as part of a long‐term partnership among the South Carolina Department of Health and Environmental Control (SCDHEC, now the SC Department of Environmental Services ‐ SCDES), the Lowcountry Alliance for Model Communities (LAMC, a non‐profit community‐based organization), the Arnold School of Public Health at the University of South Carolina (USC), the Center for Coastal Environmental and Human Health at the College of Charleston (CofC), and the College of Behavioral, Social and Health Sciences at Clemson University (CU). EJ Strong was funded by the U.S. Environmental Protection Agency (EPA) via a State Environmental Justice Cooperative Agreement grant. Staff, graduate students, and interns from all partner institutions and some community leaders constituted an EJ Strong Core Team that met almost weekly over the program duration. In addition, the Community Engagement Core (CEC) of the National Institute of Environmental Health Sciences (NIEHS) funded Center for Oceans and Human Health and Climate Change Interactions (OHHC^2^I) at USC contributed important support.

The overarching goal of the EJ Strong pilot program was to empower participating EJ communities and strengthen their resilience by helping them build capacity to better prepare for, respond to, and recover from disasters. The training was provided for adults and focused on types of hazards and disasters that were of primary concern to the communities, namely hurricanes and other major storms, flooding, chemical releases, and wildfires, and how all of these may be worsened by climate change. To the best of authors' knowledge, this is the first use of CMDRR training materials in the U.S.

Specific program objectives were to:

1. Construct a holistic CMDRR curriculum for use with U.S. EJ communities, based on materials developed by The International Institute of Rural Reconstruction (IIRR) and Cordaid (Asia & Cordaid, 2013) and used successfully in the Philippines and elsewhere.
2. Conduct pilot CMDRR training activities with EJ communities in SC, centered on communities in Charleston and North Charleston.
3. Focus training on hazards of special concern in SC, including hurricanes, flooding, chemical releases, pandemics, and others as may be identified by communities.
4. Apply the CMDRR curriculum in select communities to implement field practicums (i.e., hazard, vulnerability, and capacity assessments) and develop action plans.
5. Make the curriculum and program materials available for others to use via recordings, publications, products, and a freely available online course.

## 2 Materials and Methods

The EJ Strong pilot program was built upon a mature partnership among SCDES, LAMC, and the three academic institutions, with LAMC playing a key role in ensuring solid community connections and building relationships based on trust and equity. In 2007, the South Carolina General Assembly created an EJ advisory committee led by SCDES (SCEJAC 2009) and SCDES was one of five agencies across the nation awarded a State EJ Cooperative Agreement. Then in 2013, the first SC Environmental Justice Leadership Forum was established. This Forum included representatives from the academic community. One characteristic of this trust‐based relationship is that LAMC and the EJ Strong partners only work in communities by invitation. Further, the program's focus on EJ and full community participation was intentional and robust.

Comunities chosen for the EJ Strong pilot, were communities already engaged with LAMC and SCDES who had been requesting significant assistance due to the pandemic. It is important to note that many of these communities were already engaged with LAMC and SCDES (along with their academic partners) prior to the pandemic, due to chronic quality of life issues, that is, water and air quality concerns. In this regard, the EJ Strong pilot was chosen by the communities themselves. Participants for the workshops were selected based on whether they were members of the communities affiliated with LAMC or the larger EJ network across the state. Due to the program's growth over the pilot period, additional communities and participants joined in their affiliation and were involved in our activities.

**Figure 1:**
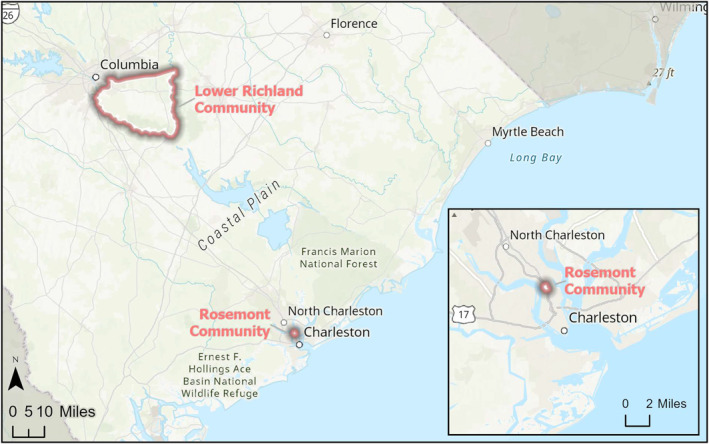
Location of South Carolina EJ Strong Pilot Communities (Lower Richland community, Columbia, SC and Rosemont neighborhood, Charleston, SC).

**Figure 2:**
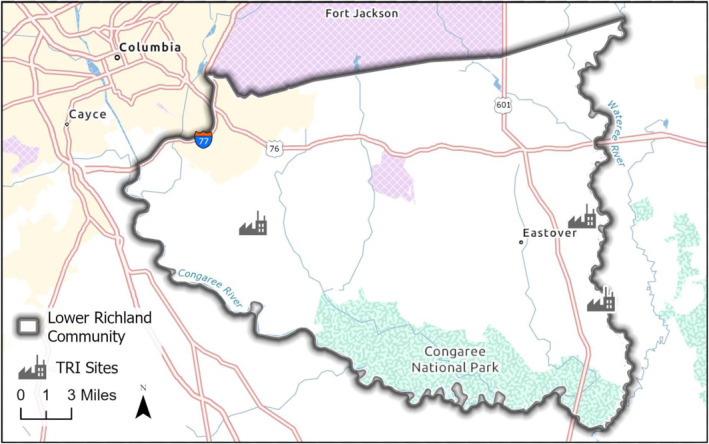
Location of field practicum community (polygon) in the Columbia Lower Richland County, near Columbia, SC. Includes listing of select USEPA Toxic Release Inventory (TRI) sites.

**Figure 3:**
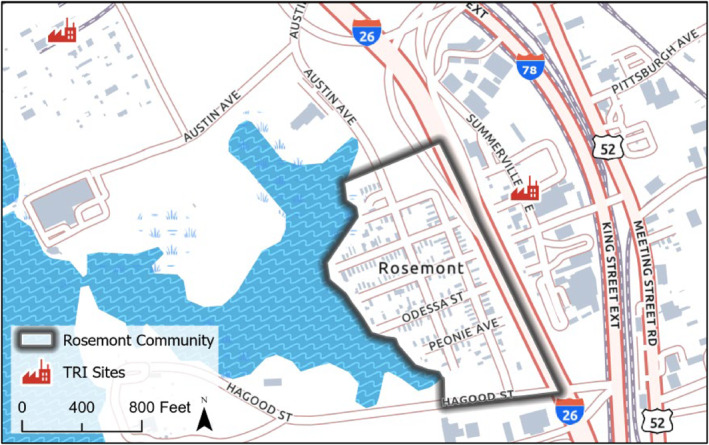
Location of field practicum community (Rosemont) in the Charleston Neck area (small red polygon and zoomed polygon). Includes listing of select USEPA Toxic Release Inventory (TRI) sites.

The primary geographic focus for the pilit was EJ communities in the Charleston‐North Charleston, SC area. These included seven communities that fall within the jurisdiction of the City of North Charleston (i.e., Accabee, Chicora/Cherokee, Five Mile, Liberty Hill, Union Heights, Windsor Place) plus one (Rosemont) that is part of the City of Charleston. Additional participants were identified through the SC EJ Hub (a network of self‐identified EJ communities across SC coordinated by the SCDES), and included communities around Greenville, Columbia (Lower Richland County[LRC]), the Pee Dee region (Pee Dee Indian Tribe), and others (Figures 1, 2, 3). Interested parties from other communities self‐identified during the pilot, and altogether one or more attendees were recorded from Aiken, Beaufort, Clemson, Columbia, Richland County, Orangeburg, Pee Dee Region (Town of McColl, Dillon County, Florence County, Marion County, Horry County), Spartanburg, St. Helena Island (Gullah/Geechee Nation), and Wateree areas in SC (Sumter County, Kershaw County, Lee County, Clarendon County and Richland County), plus Atlanta and Augusta, GA, Portsmouth, VA, Prince George's County, VA, and Washington, DC.

The Charleston area communities are burdened with multiple National Priority List Superfund sites, past and current toxic releases from industrial operations, regulated air discharges, proximity to interstate and other major highways, and a variety of legacy environmental issues, as well as challenges associated with a new port terminal with a new road, existing and new rail, and increased traffic and associated pollution (Taylor et al., 2022). Other SC EJ communities, such as those in LRC in the central part of the state, are encumbered by similar negative environmental inheritances, and these legacy conditions are exacerbated by continuing effects of the COVID‐19 pandemic, accelerating impacts of climate change, the low‐income, low‐wealth and aging characteristics of their populations, and ongoing development and gentrification.

Training Curriculum: The base curriculum chosen was the series of manuals developed and used in the Philippines and multiple countries by the IIRR and Cordaid (2013) entitled “Building resilient communities. A training manual for community managed disaster risk reduction.” The curriculum includes four booklets: (a) CMDRR Training, Design and Implementation; (b) CMDRR Concepts, Principles and Practices; (c) Facilitating CMDRR Methods and Processes; and (d) Sustaining CMDRR. These manuals encompass 12 training modules, and while some elements of all modules were included in our training activities, we emphasized materials from Modules 1 and 4–12 (Table 1).

**Table 1:** List of Modules Included in the IIRR Cordaid CMDRR Training Manuals

Module 1	Concepts, principles, and practices in CMDRR
Module 2	Evolution of CMDRR and Community Resilience
Module 3	Linking ecosystems management, climate change adaptation, and conflict to DRR
Module 4	CMDRR Framework, methods, and processes
Module 5	Introduction to Participatory Learning and Action (PLA) and PLA Toolbox
Module 6	Hazard Assessment
Module 7	Vulnerability Assessment
Module 8	Capacity Assessment
Module 9	Disaster Risk Analysis
Module 10	Community Action Planning for CMDRR
Module 11	Community Led Monitoring, Evaluation, and Learning
Module 12	Sustaining CMDRR

We adapted and modified the materials to better fit the characteristics and needs of the SC communities. We used the manuals as the foundation for preparing our own workshop and virtual training manuals which, over time, included references, graphics, and activities that culturally and ethnically aligned to U.S. and SC communities and were less patriarchal in tone. Importantly, we substantively adapted a field practicum (FP) element from the original curriculum to better fit local situations. In our FPs, material participants learned in workshop and classroom settings was put to practical use in identifying, prioritizing, and characterizing hazards, vulnerabilities, and capacities in their community. Other added elements were discussions on healthy homes, mold issues related to flooding, food security/insecurity and how to find emergency food supplies before, during, or after a disaster, mental health, connecting with emergency managers, need for additional emergency shelters, and policy and advocacy. The four EJ Strong Workshop and two FP manuals, along with other program materials, are available at https://osf.io/89gxs/?view_only=602b689115774b938cfa47cf45db6aa3.

Additionally, the materials from workshop one were developed into a freely available online course. The EJ Strong core team hired a web‐based course developer to adapt the manual from workshop 1 into an online course that future community members could engage with and use as a training resource. The core team worked with the developer during weekly meetings to create and review the content. This process took the final 6 months of the pilot period. The team chose to host the course on Moodle, a learning management platform made for web‐based education. The EJ Strong core team intends to develop workshops 2–4 into online courses using the same process, as future funding allows. The course for workshop 1 can be accessed at the following link: https://ej‐strong‐learning.moodlecloud.com/?redirect=0.

Pilot Learning Objectives: The wording of learning objectives was modified somewhat as the program got underway to better reflect expectations about what participants would understand and be able to do at completion of training activities (Orr et al., 2022). These objectives and their outcomes were developed by the EJ Strong Core Team during weekly meetings leading up to the first workshop and shortly after. Thus, they were developed among the team, then tested over the first workshop and refined afterward.

Through the EJ Strong Program, participating individuals and communities were expected to:

1. *Learn* how to identify and prevent disaster‐related environmental and health effects, especially for but not limited to flooding;
2. *Conduct* disaster planning and capacity building and identify vulnerable populations;
3. *Develop* skills to identify and direct resources needed before, during and after a disaster;
4. *Increase* individual and community resilience by putting new knowledge into action;
5. *Use* hands‐on exercises to practice response, recovery, and mitigation activities;
6. *Identify* additional skills and information needed for team captains to maintain leadership roles;
7. *Evaluate* ways to mobilize after a disaster, with emphases on healthy houses (mold), food deserts/supply, and home energy use/weatherization;
8. *Compile* best practices and lessons learned; and
9. *Define* additional training needs, if/as applicable.

Outcomes were evaluated based on the level of success of individuals and communities in identifying and prioritizing hazards of primary concern; completing hazard, vulnerability, and capacity assessments, including via FP exercises; and compiling information into community risk analyses and community disaster prevention action plans.

*Implementation*: Training was carried out via workshops, virtual mini‐workshops, FPs, and community meetings. These were highly interactive, with multiple‐way exchanges of information (e.g., between and among participants from different communities and with the EJ Strong Core Team) and incorporated local/Indigenous and scientific knowledge. Community members participated in the learning activities and helped lead them. While the EJ Strong Core Team, with participation by community members, provided draft plans and agendas for workshops, mini‐workshops, and other meetings, these were always modified by participants to ensure that issues of concern were addressed.

The initial program plan called for four in‐person workshops, augmented as needed with special topic virtual mini‐workshops. The first three workshops were intended to address the foundation and application of CMDRR, while the fourth was to focus on training community members to be CMDRR trainers going forward. The program began on 1 October 2020, and the Program Core Team had to immediately modify the training plan to accommodate the COVID‐19 pandemic emergency and its unknown duration. We postponed the initial workshop until June 2021 and adopted a hybrid (i.e., having in‐person and virtual participants) workshop format and substantially increased the number of virtual events. Altogether, we conducted four two‐day workshops in hybrid format, 11 virtual mini‐workshops, two FPs, one action plan, one hazard assessment survey, and a few other miscellaneous meetings (Table 4). Participant data were collected for each workshop, mini‐workshop, and meeting. Participants in workshops One and Four and some of the mini‐workshops were asked to evaluate these activities following the event. Despite the challenges of COVID‐19 and the resultant need to extend the program's timeline, excellent and enthusiastic community participation was recorded for every activity and throughout the program. Workshops and mini‐workshops were recorded for later review and use, as desired by community members and the Core Team, and are archived by SCDES.

The methodology for carrying out FPs and APs is well documented in our instructional “how to” videos. Short “how to” videos were recorded to help participants navigate the assessment processes for hazards, vulnerabilities, capacities, and disaster risk analysis. These are publicly available (see Text S1 in Supporting Information S1). These videos were used as instructional tools in workshops and made available to anyone interested. Included are facilitator questions to be asked for each assessment. For example, when addressing capacity readiness gaps at the community level during the capacity assessment, questions related to transportation, health and medical services, early warning, etc. are asked. Then, using the templates provided, answers to these questions are populated into the template and the risk analysis ensues. FPs were held in the Rosemont and Lower Richland County communities, and a less extensive field hazard assessment was conducted in St. Helena on the southern coast of SC. The FP activities fed directly into more comprehensive disaster risk analysis and ultimately into preparation of detailed community action plans. These communities were chosen for an FP as they acknowledged a need for this during the first workshop. Their communities were ready and willing to make these activities a priority for them during the pilot period. FPs and APs were only completed when the community made the request. The templates for each assessment are included in the data repository. EJ Screen maps used during the vulnerability assessment for Lower Richland are provided in Supporting Information S1 (Figures S1–S3).

To maintain fidelity to the original CMDRR curriculum, we attempted to keep the essentials and thus communities would not be able to customize which portions of the training they received in the workshops. However, communities were able to customize their respective field practicums and action plans per their hazard priorities. They could choose which hazards to prioritize and whether they wanted to move to the action plan phase following the field practicum. Some community members participating in the workshops chose to opt out of the field practicums during the pilot period. They received the training, however they are planning to implement at a later period in time per their specific needs and priorities.

## 3 Results

*Delivery Methods*: Workshops and other meetings with in‐person components were hosted in community facilities by community members and included virtual participants. Some of the virtal mini‐workshops included community locations for virtual participation as well as individual links. Approximate numbers of community and EJ Strong Program Core Team participants for each meeting are presented in Tables 2 and 3. Confirming counts was occasionally difficult due to participant uses of multiple mobile devices and/or attending part in‐person and part online. The numbers given are estimated to be accurate ±1–2 participants in each category. Numbers of participants in hybrid and in‐person activities ranged from 14 to 30 community members and 6–19 program Core Team members, including students and invited speakers. Mini‐workshop participation ranged from 7 to 32 community and 7–14 team members. All events included community members in planning and execution, with some led by community members. The final workshop was dedicated as a “Train the Trainer” effort where participants reviewed CMDRR fundamentals and demonstrated competence in CMDRR. Overall, the program engaged over 150 different individuals in one or more of its activities.

**Table 2:** Numbers of Participants by Category (Community Member or Project Team) in Hybrid Workshops, Field Practicums, and Action Planning Meetings

	Participants	Participants	Participants	Participants	Participants	Participants	
	Community	Community	Community	Team	Team	Team	Grand
	In person	Virtual	Subtotal	In person	Virtual	Subtotal	Total
Wkshp 1	19	11	30	10	8	18	48
Wkshp 2	10	8	18	15	4	19	37
Field Pract. 1	19	0	19	12	0	11	30
Field Pract. 2	23	0	23	9	0	9	32
Action Plan LR	23	0	23	6	0	6	29
Wkshp 3	11	3	14	10	3	13	27
Wkshp 4	24	10	34	8	4	12	46

**Table 3:** Numbers of Participants in Virtual Mini‐Workshops (All Virtual)

Mini workshop number	Community members	EJ strong team	Total
1	14	14	28
2	10	9	19
3	10	9	19
4	8	11	19
5	9	11	20
6	17	10	27
7	10	10	20
8	32	9	41
9	9	10	19
10	7	7	14
11	10	9	19

The initial Core Team was composed of nine senior staff from the five partner institutions. As the program progressed, six students (3 PhD, 2 MS, 1 MPH) were added, and several community members became engaged to the extent that they became members of the Core Team. Additionally, five undergraduate students from the Georgia Institute of Technology (GA Tech) accompanied their mentor at different times and participated in several of the workshops and mini‐workshops, took part in the training exercises, and worked with other students on campus to develop a mobile app that could be used by community members to conduct DRR assessments. These students are included in the community participant tallies for the respective program activities they were present. Also, SCDES had three undergraduate interns who assisted during 2021, and Clemson University engaged a variable number of students in its food security map work. These students were not included in the counts.

A brief description of each workshop, virtual mini‐workshop, field practicum and action planning meeting (in chronological order) is provided in Supporting Information S1 (Text S1). Table 4 provides an overview of the program timeline, focus, and results.

**Table 4:** Chronological List, Description, and Outcomes of EJ Strong Community Hybrid Workshops and Virtual Mini‐Workshops

Meeting type	Date	Focus	Results
Workshop 1	25–26 June 2021	Introduction to EJ Strong	Instruction on CMDRR principles & methods; 48 total participants
Mini‐workshop 1	25 August 2021	Hazard Assessment	Detailed instruction and “how to” conduct hazard assessments; 28 total participants
Mini‐workshop 2	13 October 2021	Vulnerability Assessment	Detailed instruction and “how to” conduct vulnerability assessments; 19 total participants
Mini‐workshop 3	3 November 2021	Capacity Assessment	Detailed instruction and “how to” conduct capacity assessments; 19 total participants
Mini‐workshop 4	1 December 2021	Community Feedback	6‐month progress review w/team & participants; 19 total participants
Mini‐workshop 5	11 March 2022	Zoning & Environmental Injustice	Instruction on the history of zoning, land use, & environmental injustice cases; 20 total participants
Workshop 2	29–30 April 2022	Healthy Homes, Food Security, & Assessments	Instruction on healthy homes, & food & nutrition security, including Clemson work on a statewide map of emergency food resources; 37 total participants
Mini‐workshop 6	1 June 2022	Rosemont FP Planning	FP planning with the Rosemont Community, 1st virtual meeting; 27 total participants
Mini‐workshop 7	7 July 2022	Rosemont FP Planning 2	FP planning with the Rosemont Community, 2nd virtual meeting; 20 total participants
Field Practicum (FP) 1	22–23 July 2022	Rosemont Community	Carried out participatory disaster risk analysis in Rosemont. 30 total participants
Mini‐workshop 8	9 September 2022	LRC FP Planning	FP planning with LRC, 1st virtual meeting; 41 total participants
Field Practicum (FP) 2	4–5 November 2022	LRC Communities	Carried out participatory disaster risk analysis in LRC. 32 total participants
Mini‐workshop 9	9 November 2022	Connecting w/Emergency Managers	State and federal Q&A with US EPA and SCDES emergency response personnel; 19 total participants
Action Plan Meeting	25 February 2023	LRC	Carried out goal setting, SMART objective develop, and activity planning in LRC; 29 total participants
Workshop 3	24–25 March 2023	Early Warning Systems	FP report outs from Rosemont & LRC; facilitated discussions & exercises on implementing early warning systems (EWS); 27 total participants
Mini‐workshop 10	27 April 2023	Mental Health & Disasters	Mental and Behavioral Health Considerations in Emergency Preparedness, Response, and Recovery; 14 total participants
Mini‐workshop 11	25 May. 2024	Emergency Policy and Advocacy	Incident command structure, what circumstances various agencies become engaged, & advocacy by non‐profit non‐governmental entities; 19 total participants
Workshop 4	28–29 July 2023	Train the Trainer	Qualifying CMDRR trainers in their respective communities; 46 total participants

*Evaluations of Workshops and Mini‐workshops*: Workshops 1 and 4 were evaluated using online surveys with Survey Monkey. We also attempted to evaluate the mini‐workshops, however responses were too few to be meaningful. Both surveys followed the same format, with some minor differences in questions.

For workshop 1, Question 1 asked whether the respondent had attended in person, virtually, or both. Questions 2–6 and 8–9 asked about levels of satisfaction overall, with workshop location, content, speakers, organization, and usefulness of the training and materials. Choices for participant responses were very satisfied, satisfied, neutral, unsatisfied. Question 7, 10, 11 solicited open‐ended responses about the value and utility of the workshop. We received completed evaluation surveys from 13 (43.3%) of the community participants. Demographic variables were not collected to maintain participant privacy and to reassure our participants that the evaluation focus of the workshops was on process improvement and satisfaction levels alone. Surveys evaluating the other workshops maintained this focus and level of anonymity. Overall, 85% of respondents reported being highly satisfied with the workshop, 13% satisfied, and 2% neutral. 75% of participants reported they were very satisfied with how useful the training materials were (25% were satisfied and 0% were neutral). Similarly, open‐ended responses were constructive and validating, with participants declaring that they learned a lot and appreciated the workshop content and presentations and especially the high levels of interactivity, engagement, and open discussion.

For workshop 4, Questions 1–8 were almost the same as for workshop 1, while Questions 9–11 and 13 were open‐ended and asked: What did you like most? What did you find most useful? How can we improve? How will you use the material? Question 12 was a yes or no question: Would you recommend this workshop for others? Results were similar to those from workshop 1. Sixteen community participants (47.0% of the total) submitted completed surveys. Again, overall satisfaction levels were high, with 90% highly satisfied with the workshop, 7% satisfied, and only 3% neutral. Responses to the open‐ended questions were mostly complimentary and reflected the EJ Strong commitment to engagement, participatory learning and action, collaborations, open discussion, networking, and more. All 16 respondents stated that they would recommend the workshop to others, and there were numerous references to the need for continuation/expansion of EJ Strong. Answers to the question related to how we can improve in the future included “No improvement to note,” “Have more sessions,” “Expand geographically,” “More field practice,” “On occasion, the audio quality and volume heard through Zoom were challenging”, “…sessions should be closer together,” among several others. How participants used the materials from the previous workshops was also asked. Responses included “I will continue to share all that I learn to my communities,” “Applied it to issues in Lower Richland,” “Planning additional field practicum in UMD.”

Survey responses for both workshops made clear the levels of enthusiasm participants had for the workshop, its content and the EJ Strong team and the practical utility they saw in what they learned, both from the workshop and the interpersonal interactions it catalyzed.

## 4 Discussion

This pilot program tested DRR applications in southeastern EJ communities in the U.S. to address both acute and chronic hazard risks. Disaster research in the EJ context is nothing new (Bolin & Kurtz, 2018; Cutter, 2012), however using community‐led efforts in a DRR framework to address EJ concerns is. To the best of our knowledge, the EJ Strong program is the first to offer formal CMDRR training focused on EJ and other disadvantaged communities in the U.S. As a pilot effort, it was evaluated as a resounding success according to survey feedback, with significant potential for application in communities across the country and elsewhere. Despite challenges posed by the COVID‐19 pandemic, the program reached around 150 people over a three‐year period, with many that remained engaged over the entire duration and 46 received certificates as trainers in CMDRR at closing of the final workshop.

While disaster risk reduction and education programs are being conducted in many countries (UNDRR, 2023), true community‐focused and community‐engaged efforts still appear to be in the minority despite many acknowledgments of the need (Hagelsteen & Becker, 2019; Haque & Fatema, 2022; Iizuka, 2020; Klonner et al., 2021; McLennan, 2020; Satizábal et al., 2022; and others). Additionally, most emergency management work in the U.S. occurs from governmentally led initiatives, where community‐led resilience efforts in DRR are nearly non‐existent and similar efforts are mostly a part of the US Hazard Mitigation Grant Program (Ji & Lee, 2021). In contrast, the EJ Strong program is not only community‐centered, its products are community‐tailored and culturally specific, targeted toward specific hazards and other concerns of each participating community. It also takes an “all hazards” approach, addressing multiple hazards in ways that are both scientific and culturally responsive. It takes a “one community at a time” approach to building disaster resilience (Ma et al., 2023). In summary, the pilot used a disasters or emergencies‐focused framework and applied this to EJ communities dealing with both acute and chronic disaster risks, compounded with other quality of life concerns, that is, air pollution, water and soil quality concerns, mold exposure, etc. In essence, its one‐size‐fits‐all relevance for communities dealing with any sort of hazard, chronic or acute, makes it highly desirable, especially for EJ communities.

Also, the CMDRR approach seeks to disrupt longstanding practices of parachute or helicopter science and drive‐by‐science, with scientists dropping into disadvantaged and/or Indigenous communities and conducting research on rather than with and on‐behalf‐of communities and taking advantage of local resources and knowledge with little acknowledgment. This practice has long been decried, but despite significant movements toward community‐engaged research over recent decades (Green et al., 1995; Israel et al., 1998; Springgate et al., 2021), the parachute and helicopter problem persists. This situation has prompted community‐engaged academics, scientific societies, and journals to promote significant changes in the ways that science and the publication of study results are done to ensure equitable sharing of credit, information, products, and remuneration with countries and communities from which materials and/or information were extracted (Hoffman‐Hall et al., 2022; “Nature Addresses Helicopter Research and Ethics Dumping,” 2022; Odeny & Bosurgi, 2022; Stefanoudis et al., 2021). As Detroit native and behavioral scientist (Williamson, 2021) stated: “The focus needs to be shifted beyond mere engagement for data collection to real partnership that emphasizes sustainability, uplifts the community, and creates an opportunity for policy, systems, and environmental change.”

Through this approach, work in communities only takes place by invitation and it strives to be engaged and inclusive, with a commitment that all results are co‐produced with, provided to, and property of the communities (Norström et al., 2020). Community members are engaged in virtually all aspects of the program, and publications like this paper include numerous community members as co‐authors.

Because “disasters are inherently social in nature” (Adams et al., 2023), robust social capital and social networks are widely recognized as important components of disaster resilience for individuals and communities (Chandra et al., 2018; Hu et al., 2022; Ma et al., 2023; Sandifer & Walker, 2018). EJ Strong emphasized the building of social linkages among participants and communities through exercises and breakout sessions and took advantage of existing relationships. Community participants regularly remarked about the feelings of community and connection generated by program activities and interactions.

Internationally, inclusion of females in DRR training and recognition of their skills and resources that can be applied to DRR are low (UNDRR, 2023). This is not the case with EJ Strong. Our workshops and mini‐workshops typically involved more women than men, and people of various ages from young (early 20s) to the elderly (early 80s). Women were well represented in the program Core Team's leadership, among participating community leaders and members, and most of the students involved were female. It is important to note that participant demographics were not explored, in particular socioeconomic status, as demographic variables for participants, for example, income level, were not included in the evaluative surveys for the workshops. A potential limitation of the representativeness of our participants, specifically for income level, is acknowledged. However, the intention of this pilot program was to engage with and first train community champions, then achieve increased reach of the program into subsequent years with the expectation that community champions continue CMDRR efforts in their communities. It was not possible to assess income level (among other demographic variables) without including this question (among others) in the surveys. Again, this was intentional to ensure participants felt their privacy was protected and the fidelity of the evaluation focus was maintained.

Although 15% of the world's population live with some form of disability, disabled and differently abled people are infrequently considered or specifically included in DRR efforts. Crawford et al. (2021) created “Person‐Centered Emergency Planning (P‐CEP)” materials for training of disabled people and their caregivers. This free, online resource is intended to help address the disproportionate impact of disaster events on the disabled and includes a workbook that can be used by to customize training to needs of individuals (www.collaborating4inclusion.org/pcep/). While we did not target disabled persons in the EJ Strong program, some participants had mobility, hearing and other issues and were able to take advantage of the training. Future iterations of our online course may consider addition of P‐CEP type materials.

The need to include information about ecosystems and ecosystem services (Reyers et al., 2015) was recognized from the beginning in EJ Strong. One of the early activities in workshop 1 was an illustration of how all parts of an ecosystem are related and how significant impact to any can result in negative consequences for people through degradation or interruption of critical ecosystem services (Sandifer & Sutton‐Grier, 2014). Ecosystem concerns were voiced frequently throughout the course, and community residents described their communities as being part of larger social‐ecological systems in Charleston (Taylor et al., 2022).

Okpodu et al. (2023) highlight the overlap between the domains of DRR, public health, environmental health, and where all three converge is EJ. Their conceptual framework details the various disciplines comprised in the EJ Strong core team, but missing is a key discipline, emergency management (EM). There is a significant need to include EM in this diagram given that disasters have highlighted the significant disproportionate impact on minority communities, that is, Hurricane Katrina, the 2004 Indian Ocean Tsunami, COVID‐19, etc. (Bakshi et al., 2022; Birkmann & Fernando, 2008; Bullard, 2018, 2018, 2018; Dyson, 2006; Telford et al., 2006). Previous FEMA initiatives like Program Impact in the 1990s (Armstrong, 2000) and Whole Community (Koch et al., 2017) had objectives that align with EJ Strong and are EM‐facilitated programs themselves. Over the 4 years EJ Strong has operated in SC, it is perplexing that few emergency managers have accepted invitations to engage in the effort. One must question why DRR has not received more application in local EM spaces throughout the U.S. The issue of strained resources and skeleton crews for emergencies and disasters may be a likely reason for the lack of engagement. What if more budget support from city, county, and state offices was given to creating trained EJ personnel inside those offices, with specific, data driven practices being established to address known problems? For example, low‐income public housing residents live in highly flood prone neighborhoods, but there do not yet exist policies or practices to ensure that they are safely evacuated before a hurricane. Similarly, our marginalized poor bear the brunt of heat island effects, often with no reliable cooling centers staffed to support a vulnerability they did not create. What kind of messaging can be created to make EJ issues politically expedient to the point that they get prioritized in government budget statements? EJ Strong is playing a role in filling this gap.

*Strengths*: The primary strength of the program was its high return for EJ communities on a small investment of federal (EPA) funds. This is evidenced by the program outputs, including detailed manuals of our workshops and field practicums, individual training videos, archived recordings of workshops and mini‐workshops, an EJ Strong DRR mobile app, the use of program outputs in municipal planning efforts and local schools, follow‐on funding well beyond the pilot award amount from EPA, and the online course. The latter is worthy of special note, since it takes much of the EJ Strong curriculum, along with accompanying graphics, videos, and case studies, and makes it available to anyone who is interested. The target audience is an average person from middle school age to the elderly, and one does not need special educational pre‐requisites to understand it nor specialized equipment beyond having access to the internet. The course can be taken by individuals or used by community groups or classes and does not require additional facilitation or interpretation. While available for individuals, the focus remains on communities that wish to increase their disaster coping capacity and resilience.

The demonstrated adaptability of the original IIRR and Cordaid curriculum materials is a significant strength. We were able to take curriculum materials originally developed for use in the Philippines and, with relative ease, modify and adapt them to apply to community audiences in the US. Our updated workshop and field practicum manuals include more culturally relevant graphics and examples and are freely available to anyone who wishes to use them. As in the IIRR/Cordaid booklets, we ask only that the sources be credited.

Although not originally designed this way, the program's COVID‐induced flexibility, offering both in‐person and virtual participation, ensured accessibility for a diverse range of individuals, and addressed potential barriers to attendance. The availability and accessibility of workshop and mini‐workshop recordings and the online course further extend inclusivity, allowing those unable to attend in person to access valuable information on their own schedule wherever they are.

The program's educational component covers a broad spectrum of topics crucial for community resilience, ranging from disaster risk analysis to mental health impacts after a disaster, and early warning systems. The FP element adapted and enhanced by the EJ Strong program is now considered an essential component of the curriculum. As demonstrated by the FPs in the Rosemont and LRC communities, they provide participants with firsthand experience, enhanced opportunities for practical application of the course materials to existing and emerging hazards vulnerabilities, and capacities, and help cultivate social connections and trust. The resulting field reports contribute to tangible outcomes, as seen with the integration of the Rosemont community report into the Resiliency Plan of the City of Charleston and in the use of report materials in successful community funding applications. For the LRC communities, the FP reports have already informed initial action plans, emphasizing the program's near‐term impact on community preparedness and mitigation efforts. The issuance of certificates in Community‐Managed Risk Reduction at the end of the program acknowledges participants' enhanced knowledge and helps empower them to contribute to disaster risk reduction in their respective places.

We contextualized and applied an international curriculum in CMDRR to U.S. EJ settings. We attempted to make the EJ Strong curriculum and project materials relevant for a domestic U.S‐based audience, with a particular focus on EJ communities. The curriculum is not intended for adaptation or customization for other EJ communities as the essential components from the original curriculum were adapted with the expertise of the EJ Strong core team (which consisted of state and federal EJ partners, academicians in environmental health, public health, emergency management, and community engagement, and a host of community champions). We acknowledge that this was a pilot project and improvement is always possible and thus welcome further collaboration with interested communities and partners to engage with EJ Strong for additional geographic and thematic transferability. Overall, the result was a product that was holistic (addressed acute and chronic hazards), geographically transferrable (relevant for a wide variety of communities), and provided a solution to address disaster risks in the U.S. as it addressed all phases of the disaster cycle (preparedness, response, recovery, and mitigation) and applied a systems approach to assess upstream factors that create downstream disaster risks.

CBOs and “connector” individuals are often critical components for successful community‐participatory programs (Palinkas et al., 2021; Springgate et al., 2021), including for DRR (Satizábal et al., 2022). The EJ Strong program has been particularly fortunate to have a highly trusted CBO, LAMC, as a key partner from its beginning. Founded in 2005 to address quality of life issues facing disadvantaged communities in North Charleston, SC, LAMC has long worked with communities that were the initial focus of the EJ Strong program. In EJ Strong, LAMC provides essential “connective tissue” to link all partners to the communities, introduce team members and students to communities, foster trust‐building, supply logistical support, and validate work of academics in the communities. An important partner with LAMC is the Charleston Community Research to Action Board (CCRAB). CCRAB plays a scientific role in many of the LAMC neighborhoods and several others to provide capacity and oversight in environmental monitoring systems, data collection, storage, analysis, access, reporting, visualization, and interpretation for the community members. Thus, LAMC provides advocacy support to its communities and CCRAB brings scientific oversight to ensure the data generated by the community are owned, documented, accessible and correctly understood.

While most members of the EJ Strong Core Team are academics, public health practitioners, and science‐based professionals, they are well‐versed in community‐participatory practices and committed to ensuring that the students they bring to the program are immersed in community engagement and take away valuable skills and lessons. The team expended considerable effort to establish community‐appropriate education materials and understand similarities and differences between a community's terminology and that of subject‐matter experts, and then tailor language to be understandable by as many audiences as possible.

A critically important strength has been the ability of EJ Strong to build on its activities with successful grant applications that provide ongoing funding to support both community‐based activities and continuation of EJ Strong's work and expansion to additional communities.

*Limitations*: The CMDRR curriculum was initially developed for in‐person use; however, the pandemic necessitated several accommodations. These included presentation of the material via a mix of virtual and hybrid workshops, acquiring the proper equipment to host virtual workshops, editing and supplementing the learning materials to allow virtual participation, adapting “ice‐breaker” and team‐building exercises to fit both in‐person and virtual formats, and replacing some graphics that participants felt were culturally inappropriate for our audiences. Also, some online participants experienced a gap in learning due to the long period between the first two workshops and the need to grasp education materials originally designed to be communicated in person. This was not surprising considering the problems experienced by school children and college students in dealing with COVID‐related online learning issues (Fahle et al., 2024; Hu et al., 2022). Also, retention of original participants became an issue; at the same time there was increasing interest among people who heard about the program after it was underway but who did not have the benefit of the training provided in workshop 1. To a substantial degree, the learning gaps in EJ Strong were overcome using the individual short training videos for each of the four assessments (hazards, vulnerabilities, capacities, and overall disaster risk), the mini‐workshops, and field practicums. Although it was planned that participants would attend all workshops (including the mini‐workshops), it was our expectation that attrition was inevitable. The strategy to overcome participant attrition and new participants attending subsequent workshops, was to review all previous workshop material at workshop 4. Participants who attended workshop 4 (the “Train the Trainer” workshop), received the essentials of CMDRR training, were tested, and then certified. Nevertheless, some struggled to develop an overall understanding of the program and its content. This will be explored with future iterations of EJ Strong per funding availability.

While the program reached many community members, both retention and sustained participation could be improved, and this is an issue that will require continued attention going forward, particularly with hybrid meeting formats becoming routine. As such, internet accessibility is a potential limitation going forward. Also, several of the products developed for the EJ Strong pilot program required technological awareness and/or accessibility, for example, the mobile app and the web‐based online course. Additional face‐to‐face workshops and trainings will always be warranted in this case. This necessitates increased funding from state, federal, and private sources to perpetuate the EJ Strong model and possibly to institutionalize or formalize local level accountability for disaster risk.

Developing relationships with other entities involved in emergency planning and response has been an important component of the program's success. However, while many connections were established, more work to build relationships with critically important organizations such as the American Red Cross, the SC Emergency Management Division (SCEMD), law enforcement, and other first responders are still needed and will be a focus of future efforts. Specifically, local law enforcement agencies who are intentional about building trust with members of their communities would be a promising area of collaboration with EJ Strong. As a start, the action plan meeting held in the LRC community included members from their Neighborhood Watch program and the meeting itself was held in the Sherrif's office. Future efforts with the program are expected to focus on communities across the southeast, including those on barrier islands, communities recovering from Hurricane Helene, and others dealing with compounding disaster risks from both technological and natural hazards.

## 5 Conclusions

The EJ Strong Program stands as a robust model for addressing environmental justice issues, combining education, community engagement, practical initiatives in capacity building, emergency planning, advocacy, and sustained financial support. To the best of our knowledge, the EJ Strong program is the first application of CMDRR training materials in the U.S. or focused on EJ communities. The production and public availability of detailed training manuals, a user‐friendly, do‐at‐your‐convenience online CMDRR course, story maps and other resources to assist communities in self‐advocacy efforts, and additional products and materials further the potential reach of the EJ Strong model for engagement of EJ communities across the U.S. and in other parts of the world. Future iterations of EJ Strong and our online course may include more information on other hazards such as oil and chemical spills, harmful algal blooms, water‐ and food‐borne pathogens, and microplastic pollution, in addition to climate change‐related increases in flooding, heat and humidity, mold, and other associated risks.

## Inclusion in Global Research

The research reported in this manuscript relied on field data collected in low‐resourced communities and involved collaborators from those communities. We acknowledge the authorship of many of these community members in the list of authors above where the Lower Richland and Rosemont Communities are indicated.

## Conflict of Interest

The authors declare no conflicts of interest relevant to this study.

## Supporting information

Supporting Information S1

Supporting Information S2

## Data Availability

Data collected during the pilot program pertained to the evaluations of the trainings by program participants. Surveys were delivered electronically using Survey Monkey (SurveyMonkey—Free online survey software and questionnaire tool, 2025). Results were generated using Survey Monkey's reporting functions. Maps were created by Clemson University's Geospatial Technology center and using U.S. EPA's EJ Screen Tool (US EPA, 2014). All project materials and related products can be found at the following data repository (Kilpatrick, 2024).
