# Supplementary material for: All Hazards Great and Small: Applying Disaster Risk Reduction to Environmental Justice Communities in South Carolina
Source: Geohealth. 2026 Mar 17;10(3):e2025GH001370. doi: 10.1029/2025GH001370 (PMC13093293; doi:10.1029/2025GH001370)
Supplement: Supplementary file 1 — Supporting Information S1 [file GH2-10-e2025GH001370-s001.pdf]

GeoHealth

Supporting Information for

All Hazards Great and Small: Applying Disaster Risk Reduction to Environmental Justice Communities in South Carolina

Daniel J. Kilpatrick[1], Keisha D. Long[2], Omar Muhammad[3], Dwayne E. Porter[1], Paul A. Sandifer[4], Karen Sprayberry[5], Katya Altman[1], Sean Briggs[2], Brooke Brittain[5], Nancy Button[12], John A. Cooper[5], Jeremy Cothran[1], Beata Dewitt[1,2], Tatiana DiSalvo[4], Herbert Fraser-Rahim[3], Chaquetta Greene[2], Gary Harris[6], Leslie H. Hossfeld[5], Kimberly Washok-Jones[1], Sheila Kimble[7], Merrie Koester[8], Nikhil Kulkarni[1], Herbert Maybank[12], Michael McGirr[9], Jude Owoh[1], Robert Reese[7], Michele Schaafsma[10], Judith Day (Taylor)[4], Heath Kelsey[11], Namita Koppa[13], Daphne Wilson* (*on behalf of all who participated in and contributed to the EJ Strong program)

[1]Arnold School of Public Health, University of South Carolina, Columbia, SC, USA.
[2]South Carolina Department of Environmental Services, Columbia, SC, USA.
[3]Lowcountry Alliance for Model Communities, North Charleston, SC, USA.
[4]Center for Coastal Environmental and Human Health, College of Charleston, Charleston, SC, USA.
[5]College of Behavioral, Social and Health Sciences, Clemson University, Clemson, SC, USA.
[6]Center for Sustainable Communities, Atlanta, GA, USA.
[7]Lower Richland Community, Richland County, SC, USA.
[8]University of South Carolina, Center for Science Education, Charleston, SC, USA.
[9]VI Forward, Bristol, VA, USA.
[10]Welltivity, USA.
[11]University of Maryland Center for Environmental Science, Annapolis, MD, USA.
[12]Rosemont Community, Charleston, SC, USA.
[13]College of Arts and Sciences, University of South Carolina, Columbia, SC USA.

Contents of this file

Text S1
Figures S1 to S3

Additional Supporting Information (Files uploaded separately)

Text S1: Supplemental Material, Text 1. *Chronological list of trainings*
Figure S1: Supplemental Material, Figure 1. *Toxic Release Points (graduated) in Lower Richland County, SC, 2022.*
Figure S2: Supplemental Material, Figure 2. *Wildfire Risks in Lower Richland County, SC, 2022.*

Figure S3: Supplemental Material, Figure 3. *Flood Risks in Lower Richland County, SC, 2022.*

Introduction

Text 1 in the supplemental material is a chronological list and description of the workshops and other training events held during the pilot period for EJ Strong.

Figure 1 is a map created using EPA's EJ Screen tool to graphically depict toxic release inventory (using graduated points by pounds released) for Lower Richland County, SC, 2022. This was used during the vulnerability assessment to facilitate discussion among community members.

Figure 2 is a map created using EPA's EJ Screen tool to graphically depict wildfire risks (national percentiles) for Lower Richland County, SC, 2022. This was used during the vulnerability assessment to facilitate discussion among community members.

Figure 3 is a map created using EPA's EJ Screen tool to graphically depict flood risks (national percentiles) for Lower Richland County, SC, 2022. This was used during the vulnerability assessment to facilitate discussion among community members.

Text S1. Supplemental Material, Text 1. *Chronological list of trainings.*

Workshop 1 (Jun. 25-26, 2021) introduced the EJ Strong program and the program Core Team and included an overview of the course, terms, evolution of disaster risk reduction, connecting ecosystem management and climate change to DRR, CMDRR method and process, participatory learning and action approach, and introduction to assessment of hazards, vulnerabilities, and capacities (EJ Strong 2021) (Suppl Fig. 1). Detailed instruction in conducting assessments was provided in virtual mini-workshops 1, 2 and 3 using our original instructional videos (available at: https://youtu.be/l1BsqnqKwB8 (for hazards); https://youtu.be/dwMgkwoVqLs (for vulnerability); and https://youtu.be/DWl9GOaG408 (for capacity)). Virtual mini-workshop 4 included community feedback and a holiday celebration while mini-workshop 5 focused on the history of zoning and environmental injustice.

The second two-day workshop, held on Apr. 29-30, 2022 (EJ Strong 2022a), covered healthy homes and food and nutrition security, including CU's work on a statewide map of emergency food resources. Much time was devoted to practical exercises on using the hazard, vulnerability, and capacity assessments and participatory learning to action (PLA) tools to develop community risk analyses (instructional video https://youtu.be/sOVtlor-zZk)

Virtual Mini-Workshops 6 and 7 were dedicated to planning for a detailed Field Practicum (FP) in the Rosemont community. This FP was conducted with the community over two days (July 22-23, 2022), guided by the EJ Strong FP Guide (EJ Strong 2022b). Community members welcomed the EJ Strong Core Team for a tour of Rosemont, enlightening the team about community history, its long legacy of environmental burdens, and numerous health and other concerns and providing opportunities to observe issues first-hand. During and following the walking tour, detailed assessments were completed for primary hazards of concern (flooding and chemical release), vulnerabilities to these hazards, and community capacity to prevent and mitigate these hazards. These assessments were then used to prepare an overall Participatory Disaster Risk Assessment (PDRA). Following the FP, the EJ Strong core team compiled the results into a Rosemont FP Report that was finalized after review by the community (EJ Strong 2022c).

Virtual Mini-Workshop 8 was dedicated to FP planning for LRC. LRC encompasses several distinct but interconnected communities, including Hopkins, Eastover, Gadsden, and East Columbia and includes two major rivers (Congaree and Wateree) and the Congaree National Park. LRC community leaders facilitated discussions about community preferences and operational details, including for a field trip to observe major potential hazards and their proximity to specific communities. The LRC FP was held Nov. 4-5, 2022, guided by a FP manual (EJ Strong 2022d). Highlights of the FP included an extensive survey of hazards in the several communities via a bus tour and a presentation about the devastating 1,000-year flood of October 2015 which severely affected residents. Assessments were completed for the three primary hazards of concern (flood, wildfire, and toxic release), community vulnerabilities to these hazards, and capacities of LRC to prevent and mitigate these hazards. The three assessments were then used to conduct an overall PDRA for the area. The EJ Strong Core Team compiled a draft report based on the discussions and recommendations that resulted from the practicum, and this report was finalized after review and modification by community members (EJ Strong 2022e).

Virtual Mini-Workshop 9 focused on opportunities and difficulties for building connections with local, state, and Federal emergency managers. This was followed by an Action Plan meeting for LRC held Feb. 25, 2023 in LRC to begin development of a DRR action plan for implementation in the LRC communities. The meeting group resulted in agreement on vision and goal statements and near-term goals for capacity building related to three primary hazards of concern.

Workshop 3, Mar. 24-25, 2023 (EJ Strong 2023a) included facilitated discussions and exercises on implementing early warning systems (EWS) to improve community resilience, plus updates from the LRC and Rosemont communities on follow-ups to their FPs and initial steps to implement their Action Plans, including securing funding and attention from political leaders. As a result of its FP, Rosemont will be included in the City of Charleston's Comprehensive Integrated Water Plan (*Charleston Water Plan (Comprehensive Integrated Water Plan) | Charleston, SC - Official Website*, n.d.).

Community members were also updated on the CU Food Access Map and other helpful resources.

At various times during program activities, concerns related to pervasive, serious, and sometimes long-lasting mental health issues resulting from disasters were voiced by participants. As a result, virtual workshop 10 was dedicated to the topic “Mental and Behavioral Health Considerations in Emergency Preparedness, Response and Recovery.” It was led by a community member with expertise as a mental health first responder.

The 11th and final virtual mini-workshop addressed emergency management policy and advocacy. Speakers described the incident command structure, when and under what circumstance various agencies become engaged, and advocacy by non-profit non-governmental entities.

Workshop 4, held July 28-29, 2023 (EJ Strong 2023b) (Suppl. Fig. 3), was the final workshop for the EJ Strong pilot program. Its primary objective was to help participants become qualified CMDRR trainers in their respective communities. The workshop was facilitated by the EJ Strong Core Team, with substantial collaboration from several community organizations and NGOs. Included were detailed reviews of key terms and concepts (e.g., hazards, disasters, capacities, vulnerabilities, resilience, disaster management and risk reduction), the use of PLA tools for identifying needs, and in planning, monitoring, or evaluating programs and programs within communities, and community-managed EWS. Also reviewed were program outcomes to date in communities and other advances within the program. As part of workshop 4 and a requirement to receive the course Certificate, participants had to successfully demonstrate good working knowledge of key elements of CMDRR. Each participant individually completed a summary exercise that tested their grasp of course fundamentals (see online repository for work products and project materials: https://doi.org/10.17605/OSF.IO/89GXS) Each person’s exercise was evaluated by an instructor, and a total of 46 who successfully completed the exercise were awarded a Certificate in CMDRR Training signed by SC DES and USC.

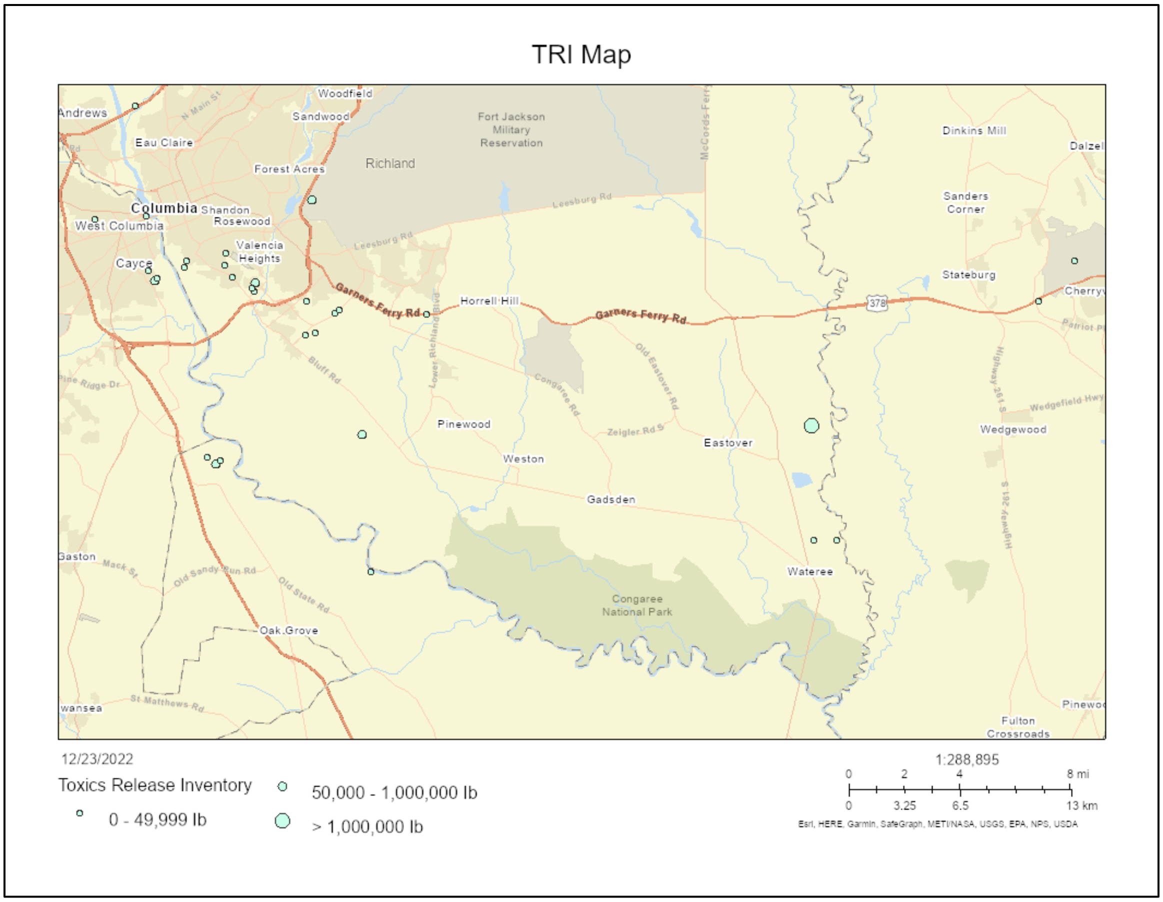

Figure S1. Supplemental Material, Figure 1. *Toxic Release Points (graduated) in Lower Richland County, SC, 2022.*

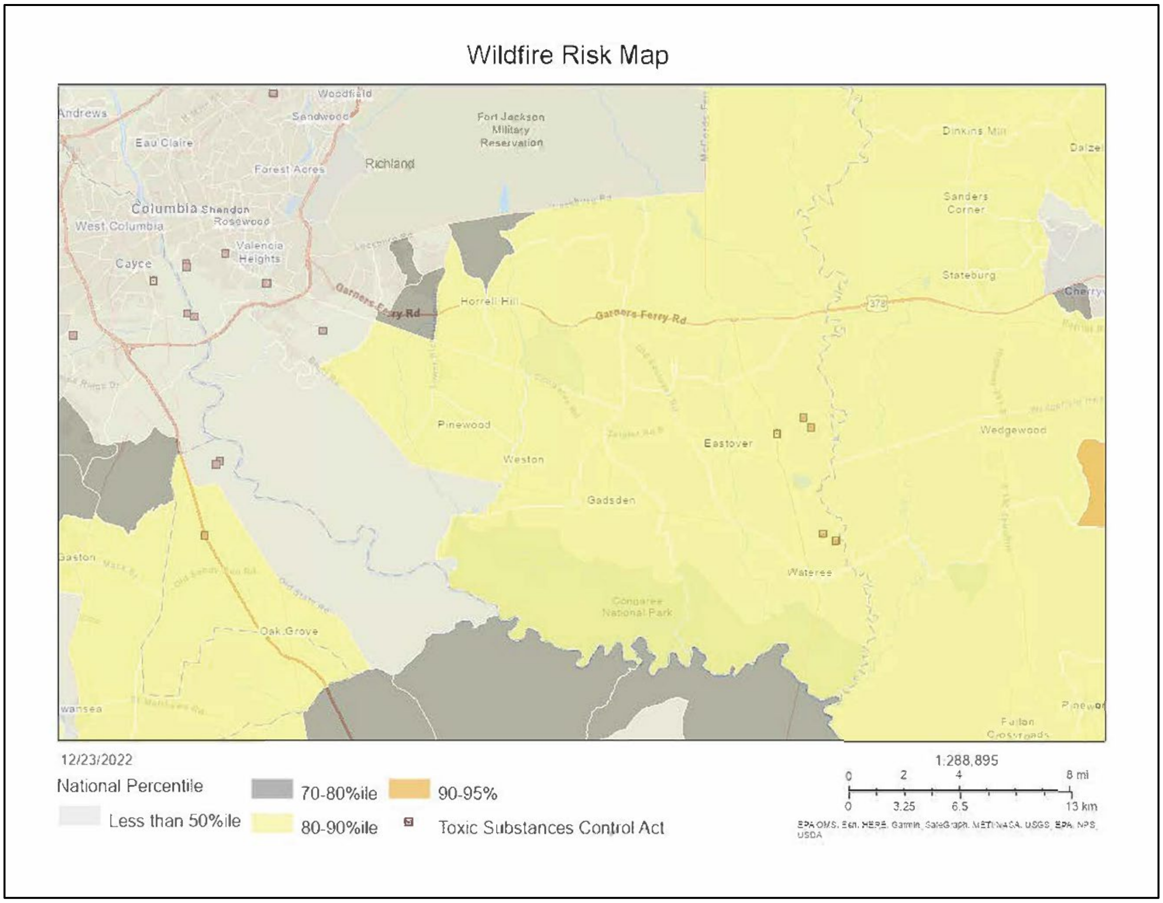

Figure S2: Supplemental Material Figure 2. *Wildfire Risks in Lower Richland County, SC, 2022.*

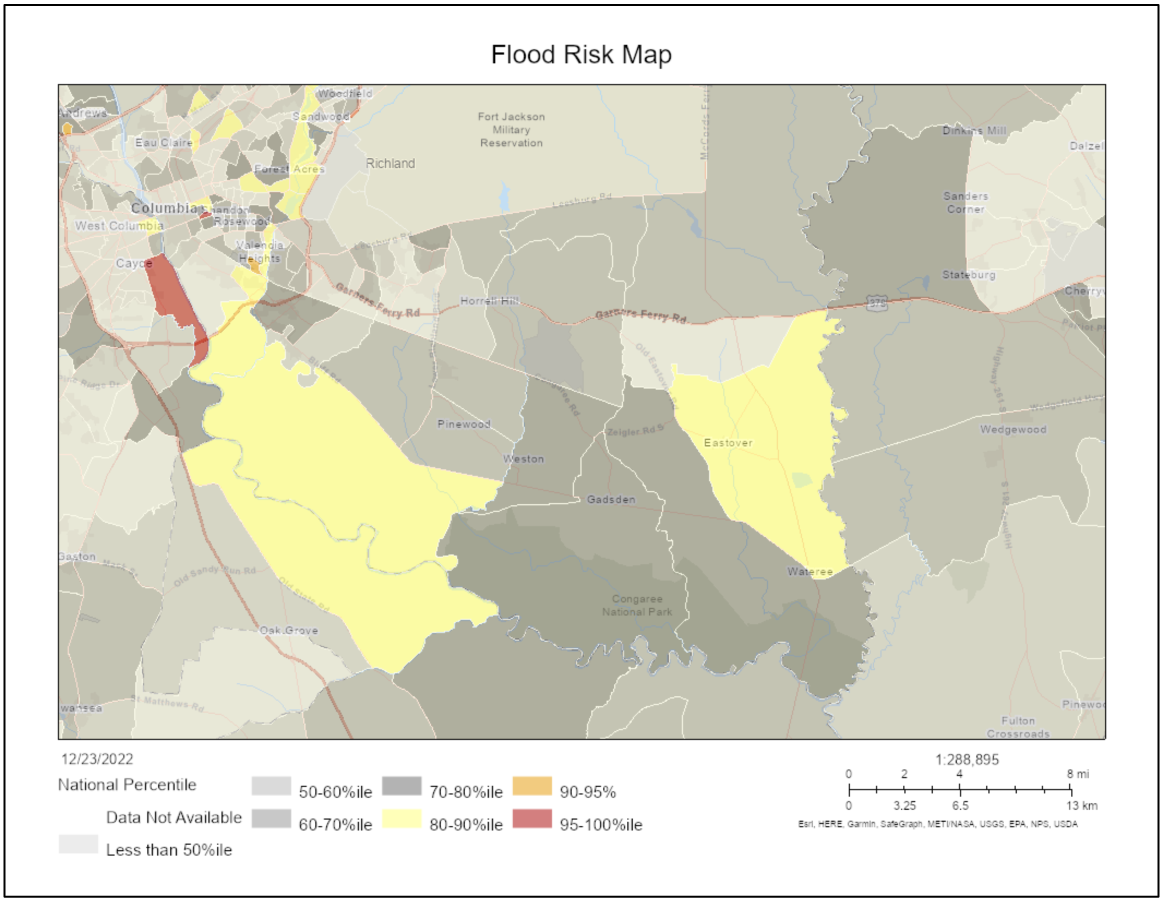

Figure S3: Supplemental Material, Figure 3. *Flood Risks in Lower Richland County, SC, 2022.*
